# “Everyone who wants to can practice on me”– a qualitative study of patients’ view on health profession students’ learning in an interprofessional clinical placement

**DOI:** 10.1186/s12909-024-05194-8

**Published:** 2024-03-08

**Authors:** Catrine Buck Jensen, Anita Iversen, Madeleine Abrandt Dahlgren, Bente Norbye

**Affiliations:** 1 Department of Health and Care Sciences, Faculty of Health Sciences, UiT The Arctic University of Norway, 9037 Tromsø, Norway; 2 Centre for Faculty Development, Faculty of Health Sciences, UiT The Arctic University of Norway, Tromsø, Norway; 3 Department of Health, Medicine and Caring Sciences, Linköping University, Linköping, Sweden

**Keywords:** Active patient involvement, Interprofessional education, Patient-centered care, Health occupations students, Medical students

## Abstract

**Introduction:**

Healthcare services face significant challenges due to the aging population, increasing complexity of health issues, and a global shortage of health professionals. Health professions education needs to adapt and develop with healthcare services’ needs. Interprofessional education and patient partnership are two trends that are increasingly being reinforced. Health professions students worldwide are expected to acquire competencies in interprofessional collaboration through undergraduate and postgraduate studies. Developing interprofessional collaborative skills in clinical placements is crucial. This study aims to explore two patients’ meetings with an interprofessional student team and better understand how the patient can participate actively in the students´ learning processes.

**Methods:**

This is a small single-case study. Two patients participated. Data was generated through participant observation and qualitative interviews. A practical iterative framework for qualitative data analysis inspired the analysis.

**Results:**

The patients observed and reflected on the interprofessional students’ learning process and felt responsible for contributing to their learning. The patients contributed to students’ learning by making themselves available for practicing and sometimes giving feedback. They considered it a win-win situation to be involved in the interprofessional learning activity as they perceived being taken seriously by the students when addressing their problems and experienced positive outcomes for their situation, such as better physical functioning and adjustments to assistive devices. Patients emphasized the importance of learning collaboration between health professionals and how this could contribute to them feeling safer as patients.

**Discussion:**

This study highlights the importance of including patients in interprofessional students’ learning processes. Patients’ active participation in interprofessional clinical placements can empower them, improve their self-efficacy, and potentially shift the power dynamic between patients and healthcare professionals. The study emphasizes the importance of the patient perspective in future research on interprofessional education in clinical settings. The study also highlights the need for clinical supervisors to facilitate patient involvement in interprofessional clinical placements and reinforce patients’ feedback for the student team.

**Concluding comments:**

Overall, this study contributes to the growing body of research on interprofessional education and patient partnership and emphasizes the importance of including patients in health professions education.

## Introduction

Healthcare services are currently facing significant challenges due to various factors, including the aging population and the increasing complexity of their health issues, global pandemics such as COVID-19, and a shift from hospitalization to community healthcare [1]. Additionally, the healthcare industry is grappling with a global shortage of health professionals [2–5].

Health professions education is closely connected to healthcare services, but it has been accused of not keeping up with the ever-shifting pace of healthcare services [6]. According to Frenk, Chen [6], health professions education needs to adjust and develop in tandem with healthcare services’ needs to prevent fragmented, outdated, and static curricula. Thibault [7] claims that health professions education is well underway as he says: “Happily, I have witnessed in the past decade a significant openness and willingness to change in health professions education with notable experimentation in both prelicensure (undergraduate) and post-licensure (graduate) education” (p. 686). He points to interprofessional education (IPE) and partnership with patients as two trends being reinforced [7].

Worldwide, health professions students are expected to acquire competencies in interprofessional collaboration through undergraduate and postgraduate studies [6, 8]. Policies and legislations in health professions education mirror this expectation [9, 10], and different competency frameworks are implemented to support the design and implementation of IPE [11–13].

A well-accepted understanding of IPE is “occasions when two or more professions learn with, from and about each other to improve collaboration and the quality of care” [14]. In interprofessional clinical placements, learning to work together is central, and IPE can enhance students’ abilities to work together and give better patient care [15]. In clinical settings, health profession students can get the opportunity to “reflect on the importance of humanizing care and the value of promoting holistic patient care” [16], and thus provide patient-centered care of better quality. Interprofessional clinical placements can improve interprofessional competencies including learning from other professions and attitude toward collaborative practice in the long term and enhance competencies in patient-oriented care [17, 18].

Patients are central to students´ learning in clinical placements, but their roles and how they are included are not always clear [19, 20]. *Active* involvement of patients in health profession education including interprofessional collaborative learning has been repeatedly emphasized throughout the past decade [21–23]. Spencer, McKimm [24] argue that students’ learning to involve patients must happen from “an early stage of training” (p. 218) and they further uphold that active patient involvement is beneficial for both students and patients. Eijkelboom, Brouwers [25] emphasize that “true patient involvement in education requires a change in the mindset of traditional educators” (p. 96). In a Norwegian context, there are legislations regulating the involvement of patients in healthcare [26] and numerous guidelines for public and patient involvement in health research [27]. Active patient involvement in health professions education is considered of value for all involved actors including patients, students, and faculty [28, 29]. From a learner’s perspective, active patient involvement contributes to numerous outcomes, for example developing students´ patient-centeredness, and professional skills as well as overcoming prejudice and stereotyping [28]. Such competencies are fundamental to ensure future patient-centered healthcare services. Despite this, there are at the time few incentives for patient involvement in health professions education.

This study aims to explore two patients´ meetings with an interprofessional health professions student team and better understand how the patient can take an active part in the student’s learning process. Two research questions will be explored: (1) What are the patients’ views on health professions students’ learning in an interprofessional clinical placement? (2) What are the patient’s views on their contribution to students´ learning in an interprofessional clinical placement?

## Methods

This study is designed as a small single-case study where data was generated as a part of a multisite collective case study in a doctoral project on the patient’s role in interprofessional education. This paper is closely related to papers published elsewhere [19, 20, 30, 31].

A case study approach allows researchers to gain an “in-depth, multi-faceted understanding” [32] of complex, social phenomena [33]. Central to case studies is “the need to explore an event or phenomenon in depth and in its natural context” [32]. In this case, we want to explore interprofessional learning with patients and the patient’s perspective of this learning process.

### Context of study

The context of the study was a specialized Norwegian rehabilitation institution offering services to people with severe illness or injury. Patients admitted to the institution are referred from regional hospitals and admitted for a limited time and complex functional impairments are common among admitted patients. Interprofessional placements are arranged annually mainly for final-year health profession students with a length of five days. An interprofessional student team consisting of five students oversaw the treatment of two pre-selected patients (See Table 1). The students provided the patients with daily care, physiotherapy, and occupational therapy. At several points during the five days, the student team had supervision sessions with the interprofessional supervisor who also oversaw coordinating the placement. The supervisor had a part-time position at the rehabilitation institution.

**Table 1:** Breakdown of student professions

Study program	Year of study	Number of students
Nursing	2nd year (third semester)	3
Occupational therapy	3rd year (fifth semester)	1
Physiotherapy	3rd year (fifth semester)	1

### Participants and recruitment

Purposeful sampling [34] was applied to recruit participants. The two patients– Carla and John (fictive names)– were considered appropriate for the students´ interprofessional learning and requested to join the interprofessional learning activity. In virtue of participating in the learning activity, they were subsequently requested to participate in the study. Carla and John received oral and written information about the study and what they could expect by participating before the clinical placement started. Carla and John were both middle-aged and cognitively healthy. They had recently suffered illness and injuries in the central nervous system that throughout the past months had caused major changes in their lives. Both were granted a stay at the rehab facilities to improve their physical functioning before returning home.

The five students were attending profession-specific clinical placement at the rehab and were included in the interprofessional student team. Moreover, when the interprofessional supervisor coordinated the placement, the students received information about the study.

Information about the study was repeated when the first author and principal investigator (CBJ) met the participants on the first day of the clinical placement.

### Data generation

The study was inspired by a focused ethnographic approach [35] where the research focus was decided before data generation started. The data includes field notes from fieldwork in the rehab and two individual interviews. Data was generated during an interprofessional clinical placement period in which a student team of five health professionals cared for two patients over five days.

### Participant observation

CBJ did participant observations of the interprofessional student team for five days, observing students´ preparations, interaction, care, and treatment of Carla and John. Also, the students´ interprofessional supervision sessions were observed. While the total observations were 32 h, observations of direct interaction between the students and patients were approximately 11 h. Occasions where students helped the patients with intimate care, such as morning routines were not observed.

### Qualitative interviews

CBJ interviewed Carla and John respectively on day three and day five of the clinical placement. Carla was unavailable for interviews on the last days of the placement, hence the interview was conducted on day three. The interviews took place in the patient’s room and were audiotaped. The length of the interviews was 42 and 36 minutes. An interview guide with open-ended questions was developed to indicate the themes of interest. Examples of themes were the patient’s understanding of the student team´s encounters with them, expectations of their meetings, and their perspective on the students´ learning outcomes of learning with and from patients.

### Data analysis

A practical iterative framework for qualitative data analysis [36] inspired the data analysis. The framework highlights the researcher’s reflexivity and “conversation” with the data. Srivastava and Hopwood [36] emphasize the importance of reflexive iteration which they claim, «is at the heart of visiting and revisiting the data and connecting them with emerging insights, progressively leading to refined focus and understandings» (p. 77). Iteration between three questions is central in the framework; What is the data telling me? (Q1) What is it I want to know? (Q2) and What is the dialectical relationship between what the data are telling me and what I want to know? (Q3). The data analysis framework does not prescribe certain steps to be followed, beyond asking the questions listed above. They highlight that articulating answers to the questions may lead to clarification and create awareness of areas of uncertainty. The authors encourage researchers to return to Q1-Q3 throughout the entire research process [36].

Our data analysis initially started throughout the fieldwork and through rewriting and structuring the field notes as well as transcribing the interviews. However, for various reasons, the data was stored for almost two years before the analysis resumed. Resuming the analysis after such a long period gave us an opportunity for re-familiarization and a wider perspective as our knowledge about the research area expanded.

CBJ took the lead in the analysis process by initially listening to the taped interviews and further reading through field notes and the transcripts of the interviews. Annotations were made along this process and initial ideas of what the data was telling (Q1) were written down. Returning to the taped interviews, listening to for example the tone of voice, were also a part of the iteration. Further iteration between Q1 and Q2 led to refinements and revisions of the research questions. For instance, the point of interest regarding active patient involvement was a result of several iterations and a response to Q1 and Q2. Refinements were discussed by the research team and additional insight was further obtained.

## Findings

Through the analysis of the interviews and the field notes, we were able to gain insight into how the patients observe the interprofessional students’ learning process and how they feel responsible and are positive and engaged in contributing to student learning. The analysis also tells us that the patients overall consider it a win-win situation to take part in the interprofessional learning activity.

### Observing the learning process

Carla and John observe and reflect on the student’s learning process, and in the interviews, they both expressed how they see the student team’s work and collaboration have evolved throughout the placement period. The individual students’ process in the team and the collaboration between the team members are considered from the patient’s perspective. For instance, John reflects on this when saying:

[…] at first, the PT student was very quiet and withdrawn but then suddenly she became very engaged and outgoing and good at telling the rest of the team what she saw and what she was doing. This was also the case with the OT student who seemed really embarrassed initially but then loosened up and it was great to see at the consultation today how he found himself in his right element when he could explain to the team [what he was doing].

Also, Carla reflects on the student team’s appearance:

The students have changed over the week, I have seen that they talk more together, in a way, yes, it seems easier for them to work together. So, it is probably quite important that the students work together like that.

Both patients related the students’ learning outcomes to their situation. Carla for example expressed how health professionals’ collaborative practice, hence what the students are in a learning process of, can make her feel safer as a patient:

I think that the best for me is that everyone in a way is harmonious or respects each other at the same rate because that makes me feel safe, it really does.

John also reflected on how the PT student following and learning from and with a nursing student in a morning routine could be important for learning about the patient:

They seem to become a little closer [with the patient] because they help you with personal hygiene, right, so I think maybe, or I’m a 100% sure, that it’s a good thing for them [PT students] to see a little more of the patient from “the bottom up”.

### Contributing to learning

Both Carla and John express in different ways how they want to contribute to students’ and health professionals’ learning. They give examples of how they make themselves available to the students for training in different procedures and how they feel that it is important to contribute to future health professionals of good quality. Carla said:

I can contribute so that the students get to practice procedures on a human, a living human, not a mannequin. I want the nurses and the physiotherapists and everyone who is undergoing health professions education to learn as much as possible, so I just said, ‘Everyone who wants to can practice on me’.

John also welcomed students to use him in training but expressed how this had been a process for him since his injury occurred:

I have worked intensely on that matter [allowing students to practice procedures on him], that´s just the way it is, they [the students] are the ones who are going to learn these skills, and if I can contribute to that it would be great.

John expresses a feeling of being responsible for giving feedback to the students. When CBJ comments that he gives feedback to the students in a concise way, he says:

I almost think that it is a part of the task that I have, it is important, that I give praise to the students who deserve it [re-written for conciseness].

In several of the consultations in the physiotherapy facilities, John also shares with the students how their treatment feels. An example of this was an occasion when he was lying on the treatment bench, surrounded by the student team. The PT student did some passive stretching on his legs, and he turned to the nursing students and explained to them how the stretching could contribute to his sleep at night [as it relieved pain and discomfort]. The nursing students immediately responded that this was new, but very important, information to them [as they were the ones who carried out stretching before bedtime].

Finally, both patients expressed that being part of the interprofessional students’ learning process had positive outcomes for their situation. They perceived being taken seriously by the students when addressing what they considered their problems to be, and John even experienced better physical functioning at the end of the clinical placement. He exemplified how not only the collaboration between the students was important but also the collaboration with the patient:

It was nice how the PT student, who apparently had good professional insight, intervened on my pain issues. Because that was the thing that I addressed as my biggest concern, and she intervened on that straight away. And that is what I mean, it must be a collaboration between the patient and the health professionals.

The pain issue that John addressed was further followed up by other members of the student team, as the nursing students suggested revising the pain medications that John had.

Carla also had some of her assistive devices at home adjusted after addressing this with the OT student. She expressed to be very pleased with the adjustments, as the devices had bothered her for a long time.

## Discussion

This paper aimed to investigate the experiences of two patients who participated in an interprofessional clinical placement with health professions students. The study seeks to understand how patients can actively contribute to students’ learning process by exploring two research questions: (1) What are the patients’ views on interprofessional health professions students’ learning? (2) What are the patients’ views on their contribution and participation in an interprofessional clinical placement?

The study’s findings suggest that patients reflect on and consider the interprofessional students’ development and find it important to contribute to their learning. The discussion section will explore the possible implications of these findings by examining four matters: (1a) the implications for students’ learning process, (b) the implications for the patients, (c) the implications for the supervisors who facilitate interprofessional learning activities, and (d) the implications for the organization of interprofessional clinical placements.

IPE can potentially enable students to develop and promote patient-centered care [37]. Thistlethwaite, Moran [38] suggest that IPE can provide students with learning outcomes on the patient’s central role in interprofessional care and their role as a partner in the interprofessional team. Interprofessional clinical placements offer an ideal opportunity to acquire such understanding [39] and practice including the patient as a partner. However, involving patients in learning activities can make them vulnerable and put them at risk [40]. Therefore, it is crucial to support patients before, during, and after a learning activity to ensure a positive experience for both patients and students [25, 40].

The patients in this study provided valuable insights into the students’ interprofessional learning process and their professional development. They expressed a willingness to make themselves available for the students and their learning opportunities, which aligns with the findings of Eijkelboom, Brouwers [25] and Spencer, McKimm [24] that patients find it meaningful to contribute to health professions education.

The patients were willing to contribute to students’ learning, and one patient even took a more active role. However, the potential for patients to contribute to learning may not have been fully realized in this setting. Other studies of interprofessional clinical placements have also shown that the patient’s perspective is often overlooked when the team plans, negotiates, and conducts their work [41].

Articulating the patient’s willingness to be involved with the interprofessional students can allow the students to practice with “low shoulders” and receive feedback from the patients on how they experienced the interprofessional student team’s actions. By involving patients more actively, students could gain a better understanding of the patient’s role in the interprofessional healthcare team and how good collaboration with the patient could contribute to better compliance or a more holistic approach. Moreover, as future health professionals, students could learn to view patients as a resource for feedback and development and adopt an approach where collaboration is central [25].

Eijkelboom, Brouwers [25] emphasize how teaching students to “ask, receive, and use patient feedback” (p. 95) can provide rich learning opportunities not only in the moment but also throughout their careers. The example where the patient gave explicit feedback in the PT facilities illustrates a valuable learning opportunity.

Assigning patients to be more actively involved, such as giving feedback to students, could become part of an active process that engages patients in their treatment. This engagement may contribute to an empowering process, where the patient can be part of the team and control what happens to them.

The Cambridge Dictionary defines empowerment as “the process of gaining freedom and power to do what you want or to control what happens to you” [42]. Although the interprofessional students in this study did not specifically aim to empower the patients, one could argue that the interaction and role that the patients had in the learning activity could make them more aware of their resources and expertise as a patient. This could potentially lead to a shift in the power dynamic between patients and healthcare professionals, where patients view themselves and are considered by health professionals as active partners in their care rather than passive recipients.

Our findings raise the question of who is responsible for involving patients or facilitating their involvement in health professions education and interprofessional clinical placements. In a previous publication, we emphasized the crucial role of the clinical supervisor in interprofessional education and how they need to shift their focus to include the patient in interprofessional clinical placements [31]. Clinical supervisors often have a clinical position and can connect the interprofessional student teams’ learning with active patient involvement.

Our findings show how one of the patients took an active role in giving feedback to the students without being prepared or asked for it, while the other patient had many thoughts on the learning process and their role as a contributor to learning but did not give feedback. We question if the supervisor could serve as a bridge builder for learning between the patients and the students. If the supervisors were more frequently present in patient-student interaction, they could help reinforce the patients’ feedback for the student team. This could take place in interprofessional supervision sessions and create a space for a meta-perspective on how the feedback can be used and what students could learn from it, both individually and as a team. Reflection on feedback together with faculty or supervisors is emphasized in other studies as crucial for self-reflection and acceptance of the feedback [25, 43, 44].

Based on our experience from several ethnographic studies, we argue that the traditions of involving patients in clinical placements still use them as a source rather than a partner in learning. Some even go as far as calling patients a learning object [45]. Our findings highlight the importance of including the patient in interprofessional students’ learning processes beyond being a receiver of team-based care in the patient role. The two patients who contributed to this study illustrate the learning potential and how the patient perspective should be emphasized in future research on interprofessional education in clinical settings.

The study´s clinical placement, involving a small, interprofessional group of students and patients, represents an ideal setting. Scaling up such placements to accommodate more students from various disciplines poses challenges, but it is achievable, as evidenced by studies on interprofessional training wards [46–48]. The key to expanding these clinical placements is fostering collaboration between healthcare services and educational institutions to align interprofessional learning outcomes. Additionally, selecting patients who are both willing and able to actively participate in the team is crucial. With proper supervision [31], this approach can enhance interprofessional collaboration, guided by patient goals, across different healthcare settings. Despite potential obstacles such as financial constraints, varying curricula, and organizational changes in healthcare (e.g., shorter patient stays) [49], the underlying principle of this study are adaptable to both small and large educational initiatives.

### Strengths and weaknesses

This paper presents the experiences of two patients who participated in an interprofessional clinical placement with students and has some methodological weaknesses that need to be addressed. Firstly, the sample size, length of interviews, and richness of data generated could be discussed. We have thoroughly discussed this matter and concluded it is a question of values and ethics. In our discussions, we have emphasized the importance of ensuring that patients who contribute to research, especially in health professions education, are valuable partners for future research and educational practice. This is not only an ethical imperative in a research context but also for us as humans. We are concerned with letting every voice be heard and guiding further development of active patient involvement in students’ interprofessional learning in clinical placements. We believe the transparency about the data basis should be sufficient to let the reader decide whether they agree with us or not.

The interprofessional supervisor and coordinator selected and invited the patients, which may have resulted in the patients being more positive about participating in students’ learning activities. Additionally, the observations were mostly conducted in consultations led by PT and OT students, which may provide a somewhat skewed picture of the interaction between students and patients. However, the first author had many conversations with the patients and the interprofessional students throughout the five days and could thus grasp more of what happened in other situations where she was not present.

Despite these limitations, the study provides valuable insights into the patient’s perspective on interprofessional clinical placements and their potential contribution to students’ learning.

### Concluding remarks

In conclusion, our research highlights the valuable insights and experiences that patients possess and how they can enhance students’ individual and team-based learning. The implications of our findings are relevant to students’ understanding of interprofessional teamwork and the role of patients as a resource for learning.

We advocate for greater patient involvement in interprofessional clinical placements, as this can empower patients, improve their self-efficacy, and potentially shift the power dynamic between patients and healthcare professionals. By involving patients more actively in students’ learning, we can also promote a more patient-centered approach to healthcare and improve the quality of care provided.

Our study emphasizes the importance of considering the patient’s perspective in interprofessional clinical placements and the potential for patients to be valuable partners in students’ learning. We hope our findings will encourage further research and practice prioritizing patient involvement in interprofessional education.

## Data Availability

The datasets generated and analyzed during the current study are not publicly available due to a lack of consent from the participants but are available from the corresponding author on reasonable request.
